# Taurine alleviates endoplasmic reticulum stress, oxidative stress, apoptosis, and glycogen accumulation induced by high glucose in the muscle cells of golden pompano (*Trachinotus ovatus*)

**DOI:** 10.1007/s42995-025-00324-7

**Published:** 2025-10-31

**Authors:** Ming-Jian Liu, Lu-Ke Zhang, Ke-Cheng Zhu, Hua-Yang Guo, Teng-Fei Zhu, Bao-Suo Liu, Nan Zhang, Dian-Chang Zhang

**Affiliations:** 1 Key Laboratory of South China Sea Fishery Resources Exploitation and Utilization, Ministry of Agriculture and Rural Affairs, South China Sea Fisheries Research Institute, Chinese Academy of Fishery Sciences, Guangzhou, 510300 China; 2 Sanya Tropical Fisheries Research Institute, Sanya, 572019 China; 3 Guangdong Provincial Engineer Technology Research Center of Marine Biological Seed Industry, Guangzhou, 510300 China

**Keywords:** *Trachinotus ovatus*, Muscle cell line, High glucose, Taurine, Oxidative stress

## Abstract

High-glucose environments induce cellular stress, particularly endoplasmic reticulum stress (ERS) and oxidative stress, in aquatic animals. Taurine, known for its cell-protective properties, has potential in mitigating such stress. This study investigated taurine’s effects on ERS, oxidative stress, apoptosis, and glycogen accumulation in golden pompano (*Trachinotus ovatus*) muscle cells under high-glucose conditions. Cells were cultured with varying glucose concentrations and taurine supplementation. Techniques including Cell Counting Kit-8 (CCK-8) assay, Nile Red staining, periodic acid–Schiff (PAS) staining, and transmission electron microscopy (TEM) were used to assess cell viability, lipid deposition, glycogen accumulation, and ultrastructural changes, respectively. High glucose increased the ADP/ATP ratio, reactive oxygen species (ROS) levels, and reduced mitochondrial membrane potential (MMP), activating AMP-activated protein kinase (AMPK). This led to glycogen accumulation via increased glycogen synthase (*gysm*) expression and decreased glycogen phosphorylase (*pygm*) expression. Taurine supplementation restored glycogen balance, reduced glucose transporter 4 (GLUT4) and peroxisome proliferator-activated receptor gamma coactivator 1-alpha (PGC-1α) protein levels, and alleviated ERS, as evidenced by reduced PKR-like ER kinase (*perk*) mRNA and glucose-regulated protein 78 (GRP78) protein expression. Also, taurine improved mitochondrial function, inhibiting apoptosis by reducing cytochrome C (CytC) release. In conclusion, taurine alleviated ERS, glycogen accumulation, apoptosis, and mitochondrial oxidative stress, providing new insights into taurine’s mechanisms and supporting its potential use as a feed additive in aquaculture.

**Supplementary Information:**

The online version contains supplementary material available at 10.1007/s42995-025-00324-7.

## Introduction

In recent years, the rapid expansion and intensification of the aquaculture industry have presented new challenges, with the fluctuating prices of fishmeal being a major issue leading to increased costs of conventional feed (Colombo and Turchini 2021). Protein resources have become a critical factor limiting the development of China’s aquaculture industry. As a crucial component of energy and carbon sources, glucose is vital for the survival and growth of organisms (Dauer and Lengyel 2019). Due to the protein-sparing effect of carbohydrates, the inclusion of carbohydrates in fish feed reduces the amount of protein ingredients required (Azaza et al. 2015). Whereas appropriate levels of carbohydrates in feed may promote fish growth and reduce feed costs, excessive carbohydrates negatively impact growth performance and physiological functions (Zhou et al. 2013). In fish, a high-glucose environment has been proven to cause oxidative stress, cellular apoptosis, glycogen accumulation, expression of inflammatory cytokines and an increase in endoplasmic reticulum stress (ERS) (Li et al. 2020; Mollazadeh et al. 2017). For example, high carbohydrate diets have been shown to induce oxidative stress in juvenile largemouth bass (*Micropterus salmoides*), thereby promoting intestinal inflammation and cell apoptosis (Zhao et al. 2021). The study on muscle cells of olive flounder (*Paralichthys olivaceus*) at the cellular level showed that incubation of muscle cells in high-glucose culture medium induces apoptosis, increases glycogen accumulation, and inhibits protein synthesis (Liu et al. 2022a). Additionally, high-glucose conditions may induce apoptosis in AF cells by promoting ERS (Pang et al. 2020).

The endoplasmic reticulum (ER) exerts a key role in various cellular functions, particularly in the folding of newly synthesized proteins and lipid synthesis (Xu et al. 2005). The ER significantly influences the formation, number, and size of lipid droplets during lipid synthesis, as well as the lipolysis of fats. ERS may directly affect lipid metabolism (Cao et al. 2014). The process of lipid synthesis is primarily catalyzed by lipogenic enzymes, whose activity is largely regulated by transcription factors, such as sterol regulatory element-binding protein 1 (SREBP1) and carbohydrate-responsive element-binding protein (ChREBP). In mice fed a high sugar diet, ERS activated SREBP 1c, promoting hepatic lipid synthesis and thus revealing the profound impact of ERS on lipid metabolism (Zhang et al. 2012). Moreover, the occurrence of ERS is closely related to cellular oxidative stress, as confirmed in both in vivo and in vitro studies, where oxidative stress was shown to trigger the ERS pathway (Ma et al. 2023). ERS combined with mitochondrial oxidative stress collaboratively leads to apoptosis in muscle cells and contributes to muscular damage (Cao et al. 2021; Rayavarapu et al. 2012). Therefore, research focusing on nutritional strategies to mitigate ERS, oxidative stress, and apoptosis is vital for enhancing the health of fish.

Taurine, also known as β-aminoethylsulfonic acid, is a sulfur-containing amino acid widely present in tissues and cells (Jakaria et al. 2019). It has shown significant therapeutic effects in various fields, particularly in treating muscular, central nervous system, cardiovascular diseases, and metabolic disorders like diabetes (Wu 2020). Taurine plays a crucial role in maintaining normal mitochondrial and ER functions. Studies in mammalian cells have demonstrated taurine’s ability to alleviate ERS, inflammation, and apoptosis (Jong et al. 2017). Particularly in high-glucose environments, taurine has been shown to effectively prevent apoptosis, primarily by improving mitochondrial function and reducing the expression of proteins associated with cell death (Xu et al. 2015; Yu et al. 2020). In the field of aquatic animals, taurine’s significance is equally evident. For example, a study involving Japanese flounder (*P. olivaceus*) revealed that dietary taurine could mitigate ER stress induced by a high-carbohydrate diet through the mediation of the PKR-like ER kinase (PERK) pathway (Zhang et al. 2021). In aquaculture, the appropriate addition of taurine to feeds has been found to enhance the growth performance of various aquatic animals (Hoseini et al. 2018; Sampath et al. 2020; Zhang et al. 2018), such as Japanese flounder (*P. olivaceus*) (Kim et al. 2005), cobia (*Rachycentron canadum*) (Lunger et al. 2007), and Senegalese sole (*Solea senegalensis*) (Pinto et al. 2010). Similarly, the application of taurine significantly mitigates the adverse effects of high carbohydrate and low fish meal diets on the metabolism and immune responses of aquatic animals (Magouz et al. 2020; Qian et al. 2021). In our preliminary dose–response experiments, we evaluated taurine at concentrations of 5, 10, 15, 20, and 25 mmol/L under high-glucose conditions, and found that 15 mmol/L was most effective in enhancing cell viability. These results provided the basis for using this concentration in subsequent experiments. Our previous studies have confirmed that exogenous taurine supplementation in juvenile golden pompano (*Trachinotus ovatus*) fed low-fishmeal diets significantly enhances growth performance, antioxidant capacity, and intestinal immunity, while also modulating the endogenous synthesis of taurine (Liu et al. 2022c). In addition, we have elucidated key transcriptional regulatory mechanisms—specifically, the roles of *HNF-4α* and *NF-κB* in activating the *cdo* gene—that govern taurine biosynthesis in *T. ovatus* (Liang et al. 2024; Ma et al. 2021). These findings provide critical insights into the multifaceted benefits of taurine supplementation, highlighting its potential as a feed additive to mitigate metabolic stress and improve overall fish health in aquaculture. However, the role of taurine in mitigating high glucose-induced endoplasmic reticulum stress (ERS) and mitochondrial dysfunction in fish muscle cells, particularly through the AMPK/PGC-1α pathway, remains unclear.

The golden pompano (*T. ovatus*), renowned for its rapid growth rate, exquisite meat quality, and ease of cultivation, has become one of the important marine aquaculture species in the southern coastal regions of China (Liu et al. 2023). Carbohydrates, due to their economic viability and availability, are widely used in aquaculture feeds (Singha et al. 2023). However, excessive carbohydrate content in feed may lead to slow growth, health deterioration, and decreased muscle quality in fish, thus limiting its application in aquaculture feeds (Zhou et al. 2022). This study aimed to analyze the molecular regulatory mechanisms at the cellular level using a constructed muscle cell model of *T. ovatus*. It investigated the regulatory mechanisms of adverse reactions in golden pompano muscle cells induced by high sugar, including apoptosis, oxidative stress, and ERS, as well as the impact and regulatory mechanism of taurine on these high sugar-induced adverse reactions in muscle cells. This research not only deepens our understanding of the responses of fish muscle cells in high sugar environments, but also provides important references for improving fish health and promoting the sustainable development of aquaculture.

## Materials and methods

### Golden pompano muscle (GPM) cell line

In this experiment, we used the GPM cell line, previously established by our research group (Zhang et al. 2024), where it was confirmed as a stable, fibroblast-like cell line derived from *T. ovatus*. The cells were cultured continuously in Leibovitz’s L-15 complete medium supplemented with 10% fetal bovine serum. The cells were maintained at a constant temperature of 28 °C in a biochemical incubator. Cells were cultured in 25 cm^2^ sterile cell culture flasks with 3–5 mL of the complete medium. Based on the cell adhesion and growth conditions, the medium was changed 8 h after cell passage. Cell growth was monitored by microscopy, and passaging was performed approximately every 2–3 days. During the passaging process, cells were gently washed three times with phosphate-buffered saline and then digested with 0.25% trypsin. The digestion was terminated with L-15 complete medium. The culture medium was slowly and gently pipetted to disperse the cells. Subsequently, based on the cell density and growth rate, the cells were passaged at a ratio of 1:2 or 1:3 and further incubated in a 28 °C constant temperature biochemical incubator.

### Glucose and taurine concentration and time selection

This study designed solutions of varying concentrations of glucose and taurine to determine the optimal effective concentrations. For GPM cells in the logarithmic growth phase, the old culture medium was discarded, and the cells were gently washed twice with PBS. The cells were then digested with trypsin solution, and the digestion was terminated with L-15 complete medium. The cells were slowly pipetted to ensure even dispersion into single cells. A 10 µL sample was taken for cell concentration determination on a cell counting plate. Based on these results, cells were uniformly plated in a 96-well plate (10,000 cells/well) with a culture system volume of 100 µL. Each experimental group was replicated at least three times under a constant 28 °C incubation condition. After complete cell adhesion, 10 µL of different concentrations of test substances were added to the wells. Immediately following the addition, the plates were gently agitated for 30 s to ensure even distribution of the reagents. The plates were then returned to the incubator at 28 °C. At predetermined time points (12 h, 24 h, 48 h and 72 h), the culture medium was carefully removed, and the wells were washed twice with PBS to remove residual reagents prior to proceeding with the assays. Glucose was set in five concentration gradients: 20, 40, 60, 80, and 100 mmol/L; taurine was also set in five concentration gradients: 5, 10, 15, 20, and 25 mmol/L. The culture plates were incubated for set times of 12 h, 24 h, 48 h, and 72 h. Cell viability was measured using the Cell-Counting Kit-8 assay according to the manufacturer’s instructions.

### Reactive oxygen species (ROS) detection and 4,6-diamino-2-phenyl indole (DAPI) staining

This utilized an ROS assay kit to measure the intracellular levels of ROS. Cells in the logarithmic growth phase were evenly plated at a density of 100,000 cells per well in a six-well plate and incubated overnight at 28 °C. The cells were then treated for 24 h with L-15 complete medium containing 10% FBS, L-15 medium with 60 mmol/L glucose, and L-15 medium supplemented with 60 mmol/L glucose and 15 mmol/L taurine. These groups were designated as control, HG (high glucose), and HG + T (high glucose + taurine), respectively. Upon completion of incubation, ROS levels in each group were assessed. Cells were incubated with 2’-7’dichlorofluorescin diacetate working solution for 20 min at 28 °C, then stained with DAPI staining solution for nuclear staining at room temperature (approximately 25 °C) for 5 min. Observations and photographs were taken using a fluorescence microscope. For detailed procedures, please refer to Supplementary materials.

### Mitochondrial membrane potential (MMP) and ADP/ATP ratio measurement

MMP changes in each group were detected using an MMP assay kit. The method of cell treatment was the same as described above, with cells being treated for 24 h according to group-specific conditions. After incubation, the culture medium was discarded, and 500 µL of JC-1 working solution was added to each well, followed by a 30 min incubation at 28 °C. The cells were then washed twice with staining buffer and replenished with complete medium for observation of fluorescence intensity under a fluorescence microscope. The ADP/ATP ratio in each group was measured using an ADP/ATP ratio assay kit. After 24 h of incubation under the respective treatment conditions, cells were processed as per the kit’s instructions.

### Cell apoptosis detection

Cell apoptosis was detected using the AnnexinV–FITC/PI double staining Cell Apoptosis Detection Kit. The method for cell treatment was consistent with the above, followed by a 24 h treatment. After collection and washing with PBS, cells were resuspended in binding buffer to achieve a concentration of 1 × 10^6^ cells/mL. AnnexinV–FITC and PI staining solutions were added, followed by incubation in the dark. Binding buffer was then added for flow cytometric analysis.

### Caspase-3 activity measurement

Caspase-3 activity was measured using a Caspase-3 Activity Assay Kit. The cell treatment method was the same as described above. After treatment, cells were washed, centrifuged, lysed, and the supernatant was collected. The protein concentration was determined using the Bradford Protein Assay Kit. Ac-DEVD-pNA was added, and absorbance at 405 nm was measured using a spectrophotometer after incubation. For details, see Supplementary materials.

### Glycogen content analysis and periodic acid–Schiff (PAS) staining

Glycogen content was measured using a Glycogen Content Assay Kit, and PAS staining was performed using a PAS Staining Kit. After treatment, cells were digested, centrifuged, and lysed, followed by boiling and centrifugation. The supernatant was used for measurement. After PAS staining, Mayer’s hematoxylin was used for nuclear counterstaining, and photographs were taken. For details, see Supplementary materials.

### Nile Red and DAPI staining

Nile Red staining was performed using a Nile Red staining kit to detect intracellular lipid content. After treatment, cells were fixed, washed, and stained, followed by observation and photography using an inverted fluorescence microscope. For details, see Supplementary materials.

### Triglycerides (TG) content measurement

Triglyceride content was measured using a triglyceride assay kit. After treatment, cells were lysed, centrifuged, and protein concentration was determined, followed by triglyceride content measurement.

### Observation of ER and mitochondrial ultrastructure in GPM cells

This was based on the methods of previous researchers with some modifications (Guo et al. 2022). After treatment, cells underwent fixation, dehydration, infiltration, polymerization, ultrathin sectioning, and staining. Transmission electron microscopy (TEM) was used to observe the ultrastructure of golden pompano muscle cells. Twenty random fields were selected for each group to observe and count the number of lipid droplets. For details, see Supplementary materials.

### Enzyme activity measurement of glycolipid metabolism

Enzyme activities of acetyl-CoA carboxylase (ACC), fatty acid synthase (FAS), hormone-sensitive lipase (HSL), adipose triglyceride lipase (ATGL), glucose-6-phosphate dehydrogenase (G6PD), malic enzyme (ME), glycogen synthase (GYSM), and glycogen phosphorylase (PYGM) were measured using biochemical assay kits provided by Jiangsu Enzyme Immune Industrial Co. Ltd. Briefly, the kit-based results were initially expressed as U/L and subsequently normalized to the cell count (approximately 5 × 10^^4^ cells per sample), resulting in a final expression of U/10^6^ cells. For details, see Supplementary materials.

### Real-time quantitative PCR analysis of mRNA expression levels

Cells were seeded at 5 × 10^4^ cells/well and treated for 24 h. Total RNA was extracted using the HiPure kit, cDNA was synthesized using the Evo M-MLV RT Mix Kit with gDNA Clean for qPCR. PCR reactions (12.5 μL) were performed with 40 cycles: 95 ℃ for 10 s, 60 ℃ for 30 s, and 72 ℃ for 30 s. RT-qPCR analysis was performed according to the laboratory’s previous methods (Liu et al. 2021). All assays were run in triplicate. Primers for genes, such as glucose-regulated protein 78 (*grp78*), *perk, srebp1,* Stearoyl-CoA desaturase (*scd*), *fas, acc, hsl,* lipoprteinlipase (*lpl*), optic atrophy protein 1 (*opa1*), mitofusin 2 (*mfn2*), *gysm,* and *pygm,* were designed using Primer Premier 6.0 (Premier Biosoft International, USA), with *EF-1α* as the internal reference gene. The gene expression level was determined using the comparative 2^−ΔΔCT^ method (Livak and Schmittgen 2001). For details, see Supplementary materials.

### Western blot analysis of protein expression levels

This was based on the methods of previous researchers with some modifications (Liu et al. 2022b). Total cellular proteins were extracted using the Thermo Scientific Mem-PER Plus Membrane Protein Extraction Kit and quantified using the Pierce BCA Protein Assay Kit, followed by SDS-PAGE electrophoresis. After transferring to a PVDF membrane, blocking, primary, and secondary antibody incubation were performed, and finally, proteins were visualized using the ECL method. The protein levels were normalized to EF-1α expression. The reagents and corresponding information used throughout the experiment are provided in the supplementary materials.

### Statistical analysis

Statistical analysis was performed using SPSS 26.0, ImageJ2, and Origin Pro 2021 for one-way ANOVA. Data normality was tested with the Shapiro–Wilk test, and homogeneity of variances with Levene’s test. One-way ANOVA was applied when both assumptions were met (*P* > 0.05). Significant differences (*P* < 0.05) were determined using Duncan’s multiple range test, and results are presented as “mean ± standard deviation” from at least three independent experiments. Other analyses were conducted using Microsoft Excel 2016.

## Results

### Selection of optimal concentrations and duration for glucose and taurine treatments

**Fig. 1:**
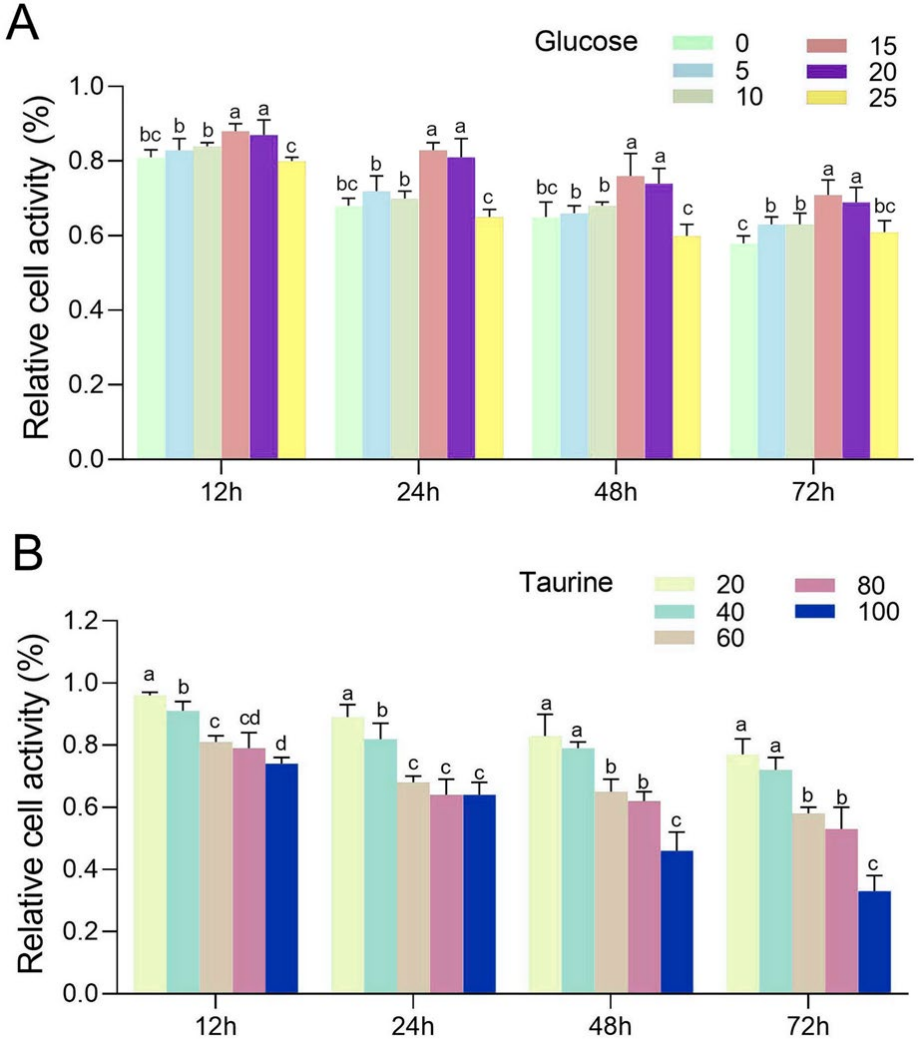
Effect of different media at various time points on the viability of golden pompano muscle cells. **A** Relative cell viability of cells incubated in media supplemented with glucose at concentrations of 20, 40, 60, 80, and 100 mmol/L at different time points. * Indicates a significant difference in cell viability between different time points (12 h, 24 h, 48 h, and 72 h) within the 60 mmol/L glucose group (*P* < 0.05). **B** Cell viability of cells cultured in high-glucose medium (60 mmol/L) with taurine at concentrations of 0 (representing high-glucose stress control), 5, 10, 15, 20, and 25 mmol/L. The 0 mmol/L group shows reduced viability due to high-glucose stress, serving as a control to evaluate the protective effect of taurine. Results are represented as mean ± SD (*n* = 3). Values with different letters indicate significant differences among groups with different taurine concentrations at the same time point (*P* < 0.05). The Y-axis represents cell viability expressed as a percentage relative to the control group

The CCK-8 assay examined the cell activity of the medium with different glucose concentrations at different time points. The results indicated that the cell viability gradually decreased with increasing glucose concentration. Cell viability in the 60 mmol/L glucose-treated group was recorded as 0.81 ± 0.02, 0.68 ± 0.02, 0.65 ± 0.04, and 0.58 ± 0.02 at 12, 24, 48, and 72 h, respectively. Compared to the 20 mmol/L and 40 mmol/L glucose treatment groups, the 60 mmol/L group exhibited a significant decrease in cell viability at all time points (*P* < 0.05). At 24 h, no significant difference in cell viability was observed between the 60 mmol/L group and the 80 mmol/L or 100 mmol/L glucose-treated groups (*P* > 0.05). However, at 12, 48, and 72 h, cell viability in the 60 mmol/L group was significantly higher than that in the 100 mmol/L group (*P* < 0.05), but not different from the 80 mmol/L group (*P* > 0.05) (Fig. 1A). Therefore, 60 mmol/L glucose was chosen as the high-glucose inducing condition. Under this condition, cell viability at 24 h significantly decreased compared to 12 h (*P* < 0.05), showed no significant difference from 48 h (*P* > 0.05), and significantly increased compared to 72 h (*P* < 0.05) (Fig. 1A). Consequently, 24 h was determined as the optimal treatment duration. With the addition of taurine to the 60 mmol/L glucose-treated group, cell viability initially increased and then decreased with rising taurine concentrations. The optimal protective effect against high-glucose conditions was observed at a taurine concentration of 15 mmol/L, thus establishing it as the best supplementation concentration (Fig. 1B).

### Effect of taurine on ROS generation induced by high glucose in GPM cells

**Fig. 2:**
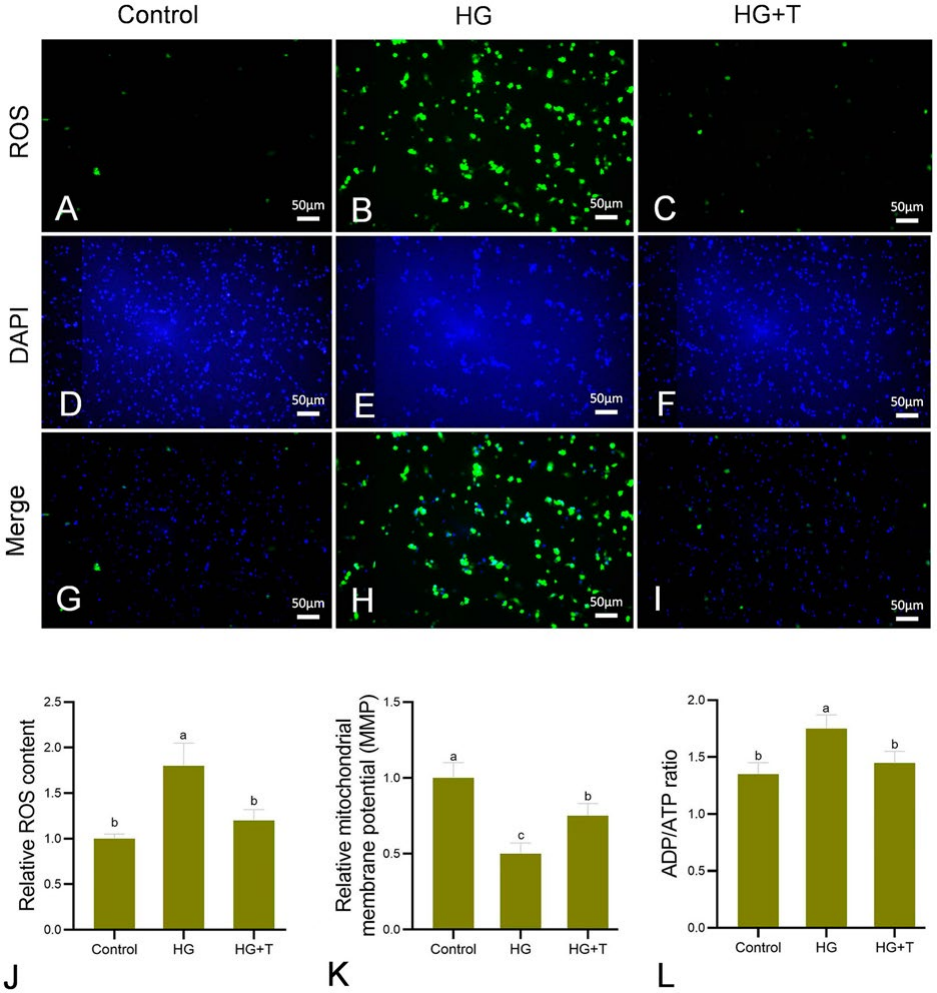
Effect of high glucose (60 mmol/L) and taurine (15 mmol/L) on intracellular reactive oxygen species (ROS) content (**A–J**), and the effects of high glucose and taurine on mitochondrial function (**K, L**), bar = 50µm. **A–I** Fluorescence micrographs of generation of ROS and 4,6-diamino-2-phenyl indole (DAPI) staining in cells. **J** Relative ROS content in cells. **K** Relative mitochondrial membrane potential (MMP) is shown by the red/green fluorescence intensity ratio with JC-1 dye. **L** Relative ADP/ATP ratio in golden pompano muscle cells. The concentrations used were 60 mmol/L glucose for HG (high glucose) and 60 mmol/L glucose + 15 mmol/L taurine for HG + T (high glucose + taurine). Results are represented as mean ± SE (*n* = 3). Values with different letters mean significant differences (*P* < 0.05)

ROS fluorescence microscopy images showed that the HG group had the strongest fluorescence signal (Fig. 2A–C), where signal intensity represents ROS content. The results clearly indicate that high glucose induces oxidative stress in cells, leading to ROS generation. Supplementation with taurine may protect the cells by reducing ROS production (Fig. 2C). The blue fluorescence represents the nuclear staining by DAPI, utilized to determine cell count (Fig. 2D–F). The merged fusion plots of the fluorescence images and the nucleostain images are shown in Fig. 2G–I. The results of ROS content in cells showed that compared to the control group, ROS levels in the HG group were significantly increased (*P* < 0.05). In the HG + T group, ROS levels were significantly reduced compared to the HG group (*P* < 0.05) and did not show a significant difference from the control group (*P* > 0.05) (Fig. 2J).

### Changes in MMP and relative ADP/ATP ratio in cells

Compared to the control group, MMP in the HG group significantly decreased (*P* < 0.05). In the HG + T group, MMP significantly increased compared to the HG group (*P* < 0.05), but was still lower than that in the control group (Fig. 2K). Compared to the control group, the ADP/ATP ratio in the HG group significantly increased (*P* < 0.05). In the HG + T group, the ADP/ATP ratio significantly decreased compared to the HG group (*P* < 0.05), and no significant difference was found between the control and HG + T groups (*P* > 0.05) (Fig. 2L).

### Effect of taurine on apoptosis induced by high glucose in GPM cells

**Fig. 3:**
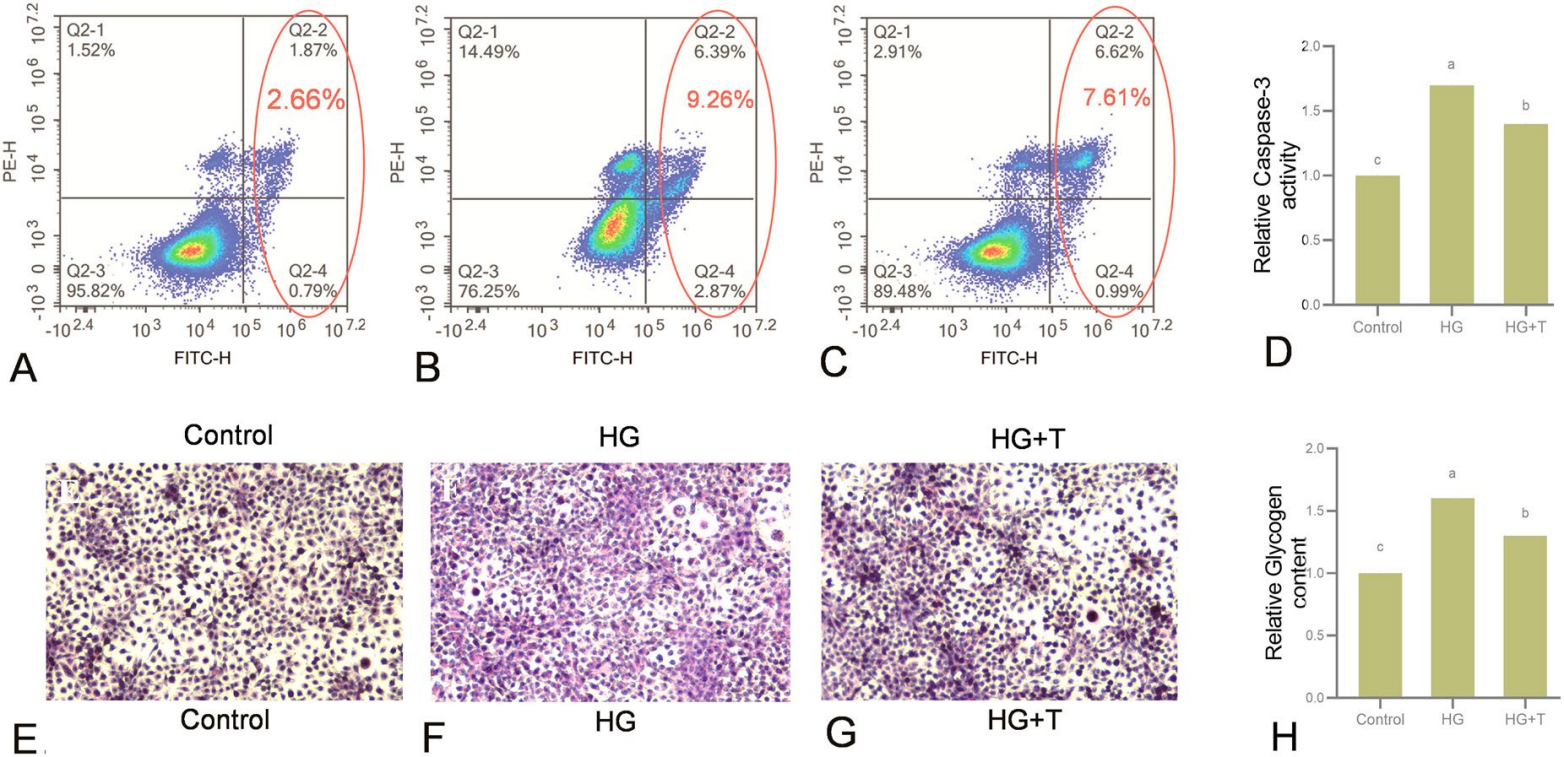
Effect of high glucose and taurine on cell apoptosis (**A**–**D**) and intracellular glycogen content (**E**–**H**). Apoptosis of golden pompano muscle cells of the control group (**A**), the HG group (**B**), and the high glucose + taurine (HG + T) group (**C**). In the circle is the proportion of apoptotic cells. **D** Relative caspase-3 activity in golden pompano muscle cells. Periodic acid–Schiff (PAS) analysis of the glycogen concentration in the control group (**E**), the HG group (**F**), and the high glucose + taurine (HG + T) group (**G**), bar = 50µm. **H** Relative glycogen content in golden pompano muscle cells. Results are represented as mean ± SE (*n* = 3). Values with different letters indicate significant differences (*P* < 0.05)

The apoptosis status of each group is represented in the four-quadrant diagram (Fig. 3A–C). After incubating for 24 h, the apoptosis rate was 2.66% in the control group (Fig. 3A) and 9.26% in the HG group (Fig. 3B). After supplementation with taurine (60 mmol/L glucose + 15 mmol/L taurine), the apoptosis rate decreased to 7.61% (Fig. 3C), indicating that 15 mmol/L taurine alleviates apoptosis induced by high glucose. This finding was further corroborated by the activity of caspase-3 (a marker of apoptosis), where high glucose in the culture medium increased caspase-3 activity. Compared to the HG group, the addition of taurine significantly reduced caspase-3 activity (*P* < 0.05) (Fig. 3D).

### Effect of taurine on glycogen accumulation induced by high glucose in GPM cells

PAS staining was used for a more visual analysis of intracellular glycogen concentration. The dark central part of the cells represents nuclear staining with hematoxylin, and the outer ring is glycogen staining. The staining results showed few cells in the control group with purple outer rings, indicating minimal glycogen accumulation (Fig. 3E). In contrast, most cells in the HG group showed extensive purple staining, indicating significant glycogen accumulation (Fig. 3F). Compared to the HG group, the HG + T group had fewer cells with extensive glycogen accumulation (Fig. 3G). The glycogen content analysis showed that the glycogen level in the HG group cells was significantly higher than that in the control group (*P* < 0.05). Compared to the HG group, the glycogen content in the HG + T group was significantly reduced (*P* < 0.05) but remained higher than that in the control group (Fig. 3H).

### Effect of taurine on lipid deposition induced by high glucose in GPM cells

**Fig. 4:**
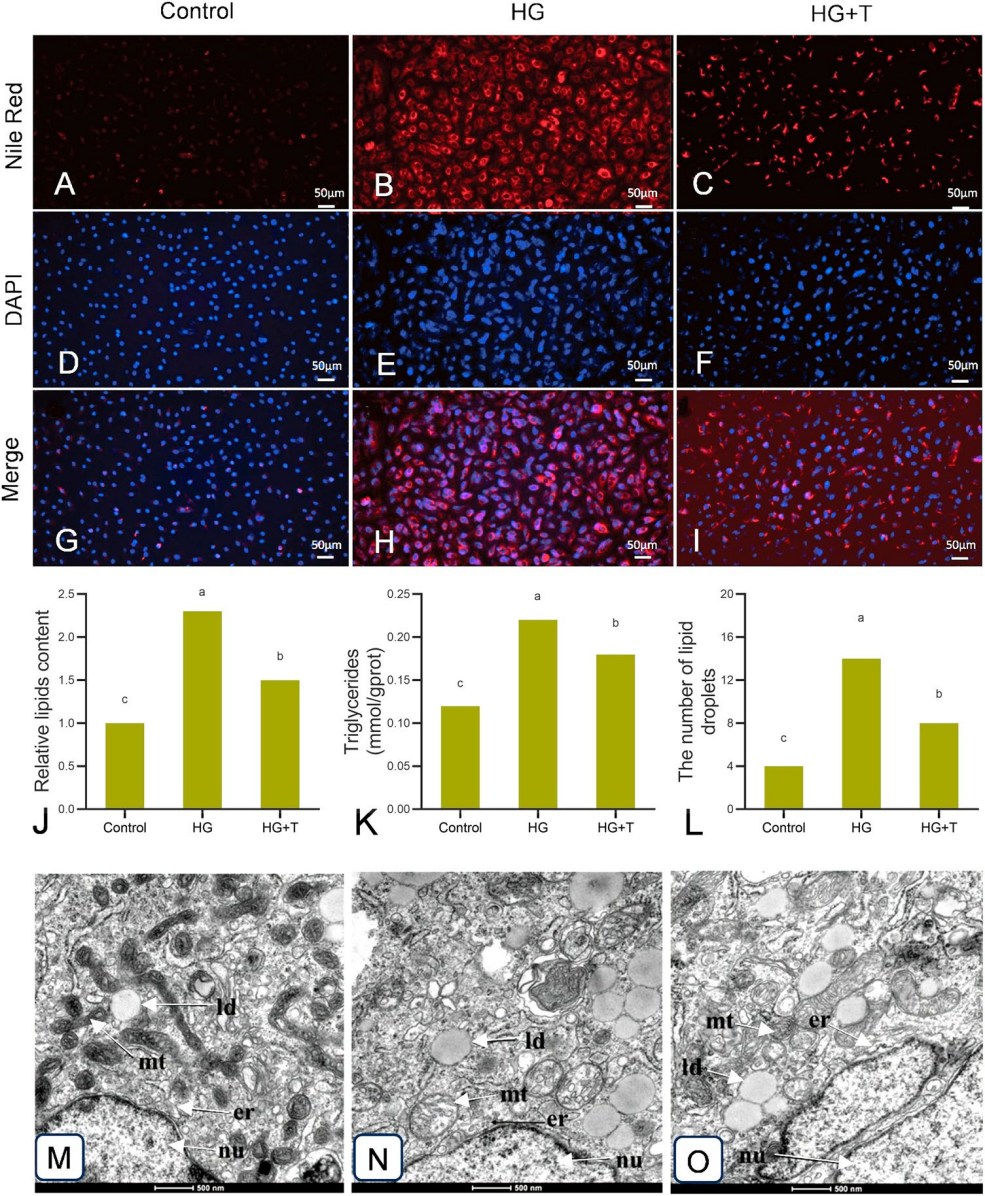
Effect of high glucose and taurine on lipids content in golden pompano muscle cells (**A**–**K**) and the transmission electron microscope images of cells (**L**–**O**). **A**-**I** Results of Nile Red staining (red: lipids) and DAPI staining (blue: nucleus), bar = 50µm. **J** Relative lipids content in golden pompano muscle cells. **K** Analysis of TG content in golden pompano muscle cells. The cells were observed under TEM. **L** Number of lipid droplets observed under TEM (20 fields observed per group). **M** Normal endoplasmic reticulum (ER) and mitochondrial structures in control group cells. **N** ER swelling and mitochondrial cristae lysis were observed in high-glucose (HG) group cells. **O** Taurine supplementation reduced ER swelling and mitochondrial cristae lysis. er, endoplasmic reticulum; mt, mitochondria; nu, nucleus; ld, lipid droplet, bar = 500nm. Results are represented as mean ± SE (*n* = 3). Values with different letters indicate significant differences (*P* < 0.05)

The Nile Red staining results for each group are shown in Fig. 4A-I. The lipid content of cells in HG group was significantly higher than that in control group, whereas that in the HG + T group was significantly lower than that in the HG group (Fig. 4A–C), suggesting that high glucose treatment increases lipid content in golden pompano muscle cells, and taurine supplementation effectively alleviates lipid deposition induced by high glucose. The blue fluorescence represents the nuclear staining by DAPI, utilized to determine cell count (Fig. 4D–F). The merged fusion plots of the fluorescence images and the nucleostain images are shown in Fig. 4G–I. The results of relative lipids content in cells showed that compared to the control group, lipids content levels in the HG group were significantly increased (*P* < 0.05). In the HG + T group, lipids content levels significantly reduced compared to the HG group (*P* < 0.05), but were still higher than that in the control group (*P* < 0.05) (Fig. 4J). Enzymatic determination of intracellular TG content revealed that the TG level in the HG group was significantly higher than that in the control and HG + T groups (*P* < 0.05). The addition of taurine led to a decrease in TG content, but it remained higher than that in the control group (Fig. 4K).

### Ultrastructural changes of ER and mitochondria in GPM cells

TEM observations of GPM cells under different treatment conditions showed ultrastructural changes of the ER and mitochondria as illustrated in Fig. 4M–O. Compared to the control group, GPM cells stimulated with high glucose exhibited altered ER morphology with swelling and a lack of clear structure, along with reduced ribosomes. Additionally, a significant accumulation of lipid droplets and mitochondrial damage, including the dissolution of cristae, was observed (Fig. 4M, N). In contrast, GPM cells supplemented with taurine showed a marked reduction in ER swelling, presence of ribosomes on the ER, decreased lipid droplets, and partial preservation of mitochondrial cristae, indicating taurine’s protective effects on the ER and mitochondria (Fig. 4O). The results of the TEM showed that the number of lipid droplets in the HG group was significantly increased compared with the control group (*P* < 0.05). The number of lipid droplets in the HG + T group was significantly decreased compared with the HG group (*P* < 0.05), but was still significantly higher than that in the control group (*P* < 0.05) (Fig. 4L).

### Enzyme activity related to glycolipid metabolism in cells

**Fig. 5:**
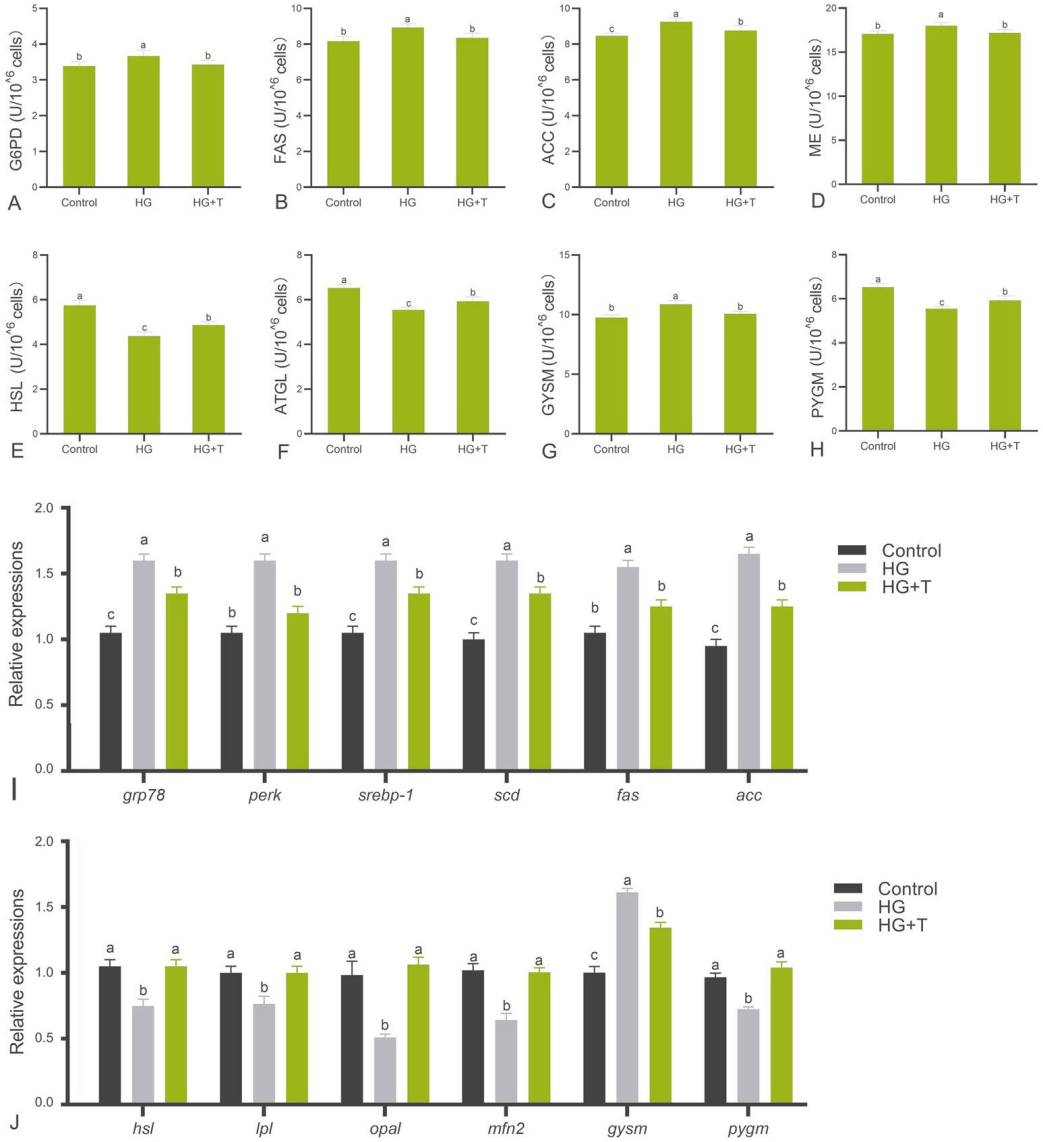
Effect of high glucose and taurine on the activity of enzymes related to glycolipid metabolism (**A**–**H**) and gene expression (**I**–**L**). **A**–**H** Results of enzyme activities related to glycolipid metabolism in control group, HG group, and high glucose + taurine (HG + T) group. **I** Relative expression of ERS response and lipid metabolism genes. **J** Relative expression of mitochondria-related genes. **K** Relative expressions of glycogen metabolism genes. Results are represented as mean ± SE (*n *= 3). Values with different letters indicate significant differences (*P* < 0.05)

The enzyme activity related to glycolipid metabolism in each group is shown in Fig. 5A–H. Compared to the control group, the HG group exhibited significantly increased activities of lipid synthesis enzymes G6PD, FAS, ACC, and ME (*P* < 0.05) (Fig. 5A–D), and significantly decreased activities of lipolysis enzymes HSL and ATGL (*P* < 0.05) (Fig. 5E, F). GYSM activity was significantly increased (*P* < 0.05) (Fig. 5G), whereas PYGM activity was significantly decreased (*P* < 0.05) in the HG group (Fig. 5H). These results indicate that HG treatment significantly enhanced the capabilities of GPM cells in fat and glycogen synthesis while reducing the capacity for fat and glycogen breakdown.

In the HG + T group, the activities of lipid synthesis enzymes G6PD, FAS, ACC, and ME were significantly lower than those in the HG group (*P* < 0.05) (Fig. 5A–D). The activities of lipolysis enzymes HSL and ATGL were significantly increased compared to the HG group (*P* < 0.05), but significantly decreased compared to the control (*P* < 0.05) (Fig. 5E, F). The activity of GYSM was significantly lower than that in the HG group (*P* < 0.05) with no significant difference from the control (*P* > 0.05) (Fig. 5G), while PYGM activity was significantly higher than that in the HG group (*P* < 0.05) and significantly lower than that in the control (*P* < 0.05) (Fig. 5H). These findings suggest that HG + T treatment significantly inhibited the increase in fat and glycogen synthesis capabilities induced by HG and ameliorated the reduction in fat and glycogen breakdown capabilities (*P* < 0.05).

### Relative expression of genes related to ERS response, mitochondria, and glycolipid metabolism

Compared to the control group, the HG group exhibited significantly upregulated expression of *grp78*, *perk*, and *srebp1* (*P* < 0.05). The HG + T group showed significantly lower expression levels of *grp78*, *perk*, and *srebp1*compared to the HG group (*P* < 0.05). The expression of *perk* in the HG + T group was not significantly different from the control (*P* > 0.05), whereas *grp78* and *srebp1* expressions were significantly higher than those in the control (*P* < 0.05) (Fig. 5I). Lipid synthesis-related genes *scd*, *fas*, and *acc* in the HG group were significantly upregulated compared to the control (*P* < 0.05), whereas the lipolysis-related genes *hsl* and *lpl* were significantly downregulated (*P* < 0.05). The expression levels of *scd*, *fas*, and *acc* in the HG + T group were significantly lower than those in the HG group (*P* < 0.05), with *scd* and *acc* remaining significantly higher than those in the control (*P* < 0.05), but no significant difference was found for *fas* (*P* > 0.05). The lowest expression of *hsl* and *lpl* was observed in the HG group, with no significant difference between the HG + T group and the control (*P* > 0.05) (Fig. 5I, J). Among mitochondrial-related genes, *opa1* and *mfn2* expression levels in the HG group were significantly lower than those in the control (*P* < 0.05), and significantly upregulated in the HG + T group compared to the HG group (*P* < 0.05), with no significant difference between the HG + T group and the control (*P* > 0.05) (Fig. 5J). Glycogen metabolism genes were also affected by high glucose treatment. *gysm* expression in the HG group was significantly higher than that in the control (*P* < 0.05), and *pygm* expression was significantly lower (*P* < 0.05), indicating intracellular glycogen accumulation. In the HG + T group, *gysm* expression was significantly lower than that in the HG group (*P* < 0.05) but higher than that in the control (*P* < 0.05), while *pygm* expression was significantly higher than that in the HG group (*P* < 0.05) and not significantly different from the control (*P* > 0.05) (Fig. 5J).

#### Western blot analysis

**Fig. 6:**
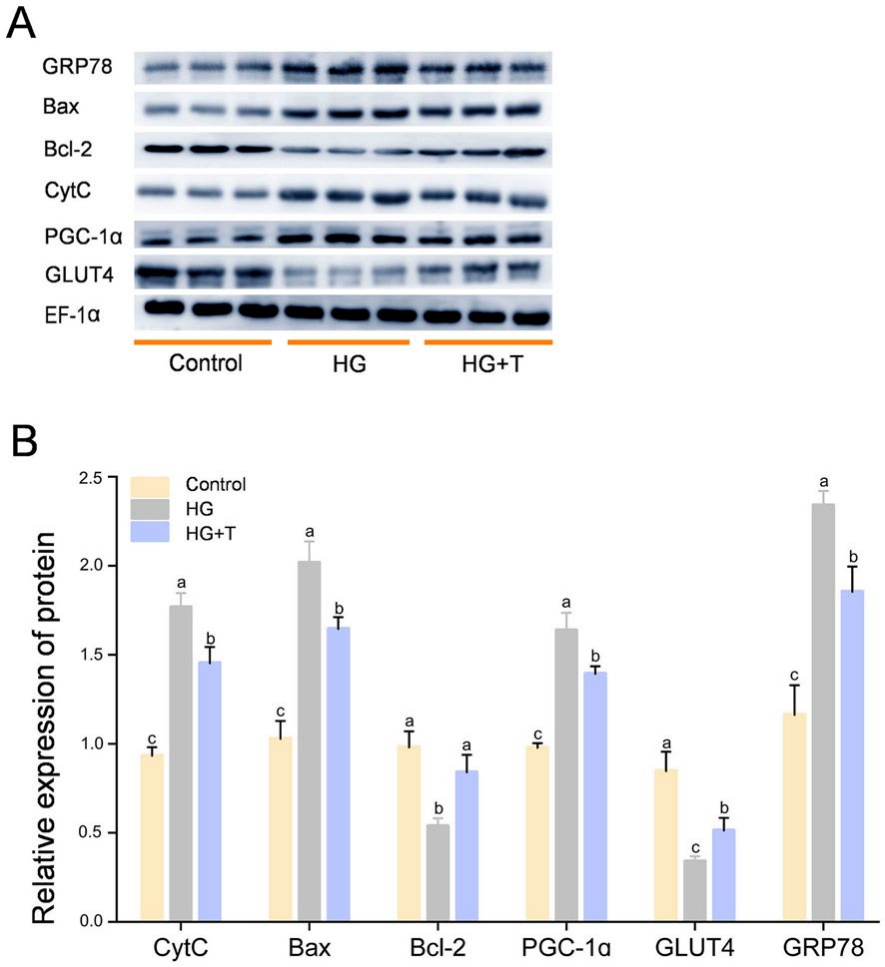
The results of Western blot analysis. **A** The expression of glucose-regulated protein 78 (GRP78), Bcl2-associated X (Bax), B-cell lymphoma 2 (Bcl-2), cytochrome C (CytC), peroxisome proliferator-activated receptor gamma coactivator 1-alpha (PGC-1α), and glucose transporter 4 (GLUT4). **B** EF-1α was used as a reference protein and the results are represented as mean ± SE (*n* = 3). Values with different letters indicate significant differences (*P* < 0.05)

As shown in Fig. 6, the study evaluated the protein expression levels of ERS factor GRP78, apoptotic factors Bax and Bcl-2, glucose transporter GLUT4, and PGC-1α in GPM cells. High glucose treatment significantly increased GRP78 protein expression compared to the control (*P* < 0.05). The HG + T group showed significantly lower GRP78 expression than that in the HG group (*P* < 0.05), but higher than that in the control (*P* < 0.05). Compared to the control, Bax expression in the HG group significantly increased (*P* < 0.05), and Bcl-2 protein significantly decreased (*P* < 0.05), indicating increased apoptosis. In the HG + T group, Bax expression was significantly lower than that in the HG group (*P* < 0.05) but higher than that in the control (*P* < 0.05), whereas Bcl-2 expression was significantly higher than that in the HG group (*P* < 0.05) and not significantly different from the control (*P* > 0.05). Cytochrome C (CytC) expression significantly increased in the HG group (*P* < 0.05), and was significantly lower in the HG + T group (*P* < 0.05) but higher than that in the control (*P* < 0.05). GLUT4, a facilitative glucose transporter, showed a significant decrease in expression under high glucose stimulation compared to the control (*P* < 0.05), consistent with glycogen concentration results, indicating intracellular glycogen accumulation and impaired transport. GLUT4 expression in the HG + T group was significantly higher than that in the HG group (*P* < 0.05) but lower than that in the control (*P* < 0.05). The HG group had significantly higher PGC-1α protein levels than that in the control (*P* < 0.05), and the HG + T group had significantly lower PGC-1α expression than that in the HG group (*P* < 0.05) but higher than that in the control (*P* < 0.05).

## Discussion

### The effect of taurine on the proliferative activity of GPM cells

The assessment of cellular proliferative activity is crucial in cell biology research, particularly in cell culture-related experiments (Zemenides et al. 2018). This evaluation not only accurately measures cellular metabolism and vitality, but also indirectly determines the impact of drug stimuli, environmental changes, and physical or chemical responses on cellular metabolism, aiding in the selection of therapeutic intervention concentrations (Dachs and Vila-Costa 2022). In this study, the CCK8 method was used to investigate the effects of different concentrations of glucose on the viability of GPM cells and to identify the optimal protective concentration of taurine. It was found that 15 mmol/L of taurine significantly enhanced the viability of GPM cells. In studies on human cervical cancer SiHa cells, taurine was observed to inhibit cell proliferation and induce apoptosis, affecting the expression of proteins such as Mammalian Sterile 20-like Kinase 1 (MST1), Bcl-2-associated X protein (Bax), and B-cell lymphoma 2 (Bcl-2) (Li et al. 2019). This suggests that taurine may have different mechanisms of action in various cell types. Additionally, a study on fish species highlighted the importance of taurine for aquatic organism development, especially during the egg and yolk sac stages, where supplementation of taurine significantly increased the growth and metamorphosis success rate of certain fish larvae (Pinto et al. 2013). These studies indicate that taurine may exhibit diverse biological effects across different organisms and confirm the complexity and specificity of taurine’s impact on cellular proliferative activity.

### The effect of taurine on high glucose-induced apoptosis in GPM cells

Beyond being the primary site of energy generation, mitochondria are also critically involved in the regulation of apoptosis (Makino et al. 2011). Mitochondrial dysfunction, particularly under oxidative stress, significantly contributes to apoptosis. A decrease in MMP is one of the causes of mitochondrial dysfunction, and previous studies have shown that high glucose treatment significantly reduces cellular MMP (Liu et al. 2022a). In this study, high glucose treatment significantly reduced the MMP in GPM cells, indicating that high glucose negatively impacts mitochondrial function. Mitochondria, being the primary source of ROS, produce more ROS when damaged, exacerbating oxidative stress. In turn, increased ROS production can cause mitochondrial dysfunction and ATP deficiency (Brownlee 2001). Previous research has demonstrated that oxidative stress causes mitochondria to consume rather than synthesize ATP, which could be one of the reasons for the increased ADP/ATP ratio under high glucose conditions (Chinopoulos and Adam-Vizi 2010). In this study, cells treated with high glucose accumulated substantial amounts of ROS, and the increased ADP/ATP ratio in the high glucose group is indicative of mitochondrial dysfunction. Taurine is essential for mitochondrial function. Studies have shown that supplementation of taurine in neuronal cells improves mitochondrial function by increasing MMP (Lambert et al. 2015). In this study, the addition of taurine in high glucose culture media significantly ameliorated the reduction in MMP caused by high glucose (Ramos-Mandujano et al. 2014).

Optic atrophy protein 1 (OPA1), mitofusin 1 (MFN1), and mitofusin 2 (MFN2) are three proteins essential for mitochondrial membrane fusion and regulating mitochondrial structure (Zhang et al. 2016). OPA1, necessary for mitochondrial function and inner membrane fusion, is associated with improved mitochondrial function and energy efficiency and may reduce ROS production by mitochondria (Robert et al. 2021). MFN2 is crucial for mitochondrial outer membrane fusion; previous studies have found that a lack of MFN2 leads to mitochondrial dysfunction and ROS production (Schisano et al. 2012). In this study, the *opa1* and *mfn2* expression levels decreased with the increasing ROS content in the HG group, consistent with the previous findings (Ramzan et al. 2020). After taurine supplementation, significant upregulation of *opa1* and *mfn2* expression levels was observed, confirming that taurine might improve mitochondrial function by reducing ROS generation and upregulating mitochondrial membrane fusion protein mRNA expression.

Excessive ROS damage to mitochondrial membranes leads to the release of CytC, which then induces apoptosis (Hüttemann et al. 2011). In this study, high glucose significantly increased the expression of CytC protein in GPM cells, while taurine significantly decreased its expression. In primary hepatocytes of Atlantic salmon, taurine has been shown to attenuate apoptosis by inhibiting the expression of pro-apoptotic proteins (Espe and Holen 2013). The activity of caspase-3 can be an indicator of the apoptosis process, and it is a downstream molecule of CytC that may be activated by CytC release (Keller et al. 2018). This aligns with the findings of this study, where caspase-3 activity significantly increased in the HG group of GPM cells, and taurine significantly reduced the increase in caspase-3 activity induced by high glucose. These results suggest that taurine inhibits the increase in apoptosis in HG-treated cells, which was also confirmed by flow cytometry analysis, showing a lower proportion of apoptotic cells in the HG + T group compared to the HG group. Similar findings have been reported in previous studies, where high glucose treatment increased apoptosis in cardiomyocytes and primary rat hepatocytes through activation of the mitochondrial-mediated apoptotic signaling pathway (Kapoor et al. 2013; Wang et al. 2018), and taurine significantly inhibited the release of CytC in PC12 cells, suppressing mitochondrial-dependent apoptosis. Therefore, the observed significant reduction in ROS production in taurine-treated cells reduces mitochondrial membrane damage, improving mitochondrial function.

### Effect of taurine on high glucose-induced lipid deposition in GPM cells

High glucose levels typically lead to cellular apoptosis, oxidative stress, mitochondrial damage, and an increase in ERS (Mollazadeh et al. 2017). The results of this study demonstrated that high glucose induced lipid deposition in GPM cells. The HG + T group showed a significant alleviation of high glucose-induced lipid deposition compared to the HG group. Transmission electron microscopy of each group’s cells revealed ER swelling and increased lipid droplet synthesis in HG group cells, typical structural changes of the ER during ERS (Zhu et al. 2012), indicating the activation of ERS during high glucose treatment of GPM cells.

Studies have shown that the ER significantly influences the formation, number and size of lipid droplets during lipid synthesis and the lipolysis of fats, directly affecting lipid metabolism (Zhao et al. 2013). Many studies report that ERS regulates lipid synthesis by controlling *srebp1*, and in mice fed a high glucose diet, ERS activates *srebp1c*, promoting liver lipid synthesis (Zhang et al. 2012). In rat pancreatic β-cells and pancreatic tissue, ERS, once activated by drugs, promotes the expression of *srebp1*, with subsequent upregulation of lipid synthesis-related genes *fas* and *acc* (Wang et al. 2005). Kammoun et al. (2009) determined that ER stress may activate *srebp1c*, thereby promoting the expression of downstream target genes, leading to lipid deposition. This is consistent with the results of this study, suggesting that ERS regulates *srebp1*, thereby activating the expression of target genes *fas*, *acc*, and *scd1*, promoting TG accumulation and lipid droplet formation, inducing lipid deposition. Previous studies have confirmed that ERS affects lipid metabolism, but the underlying mechanisms of SREBP1 activation by ER stress have been poorly investigated (Kim et al. 2018). It has been shown that SREBP 1c may activate the expression of genes related to fat synthesis through nuclear translocation, thereby regulating fat formation (Peterson et al. 2011).

In this study, after high glucose treatment of GPM cells, significant upregulation of the unfolded protein response (UPR) pathway marker genes *grp78* and *perk* was observed, indicating the activation of the UPR pathway during lipid deposition in golden pompano muscle cells. The high glucose treatment altered cellular homeostasis, leading to the accumulation of unfolded proteins in the ER, thereby activating the unfolded protein response and ERS (Wan and Jiang 2016). The classical UPR pathway activated by ERS has been shown to have different regulatory roles in mammalian fat and liver lipid metabolism (Han et al. 2013). However, studies on the impact on fish lipid metabolism are scarce. For example, research on yellow catfish has shown that the ERS-activated CAMP/PKA pathway may induce lipolysis in hepatocytes (Song et al. 2017). In *Symechogobius hasta* (*Temminck et Schlegel*), the UPR pathway regulates hepatic lipid deposition by activating SREBP 1c (Song et al. 2016). In this study, the *grp78* /*perk* pathway was considered as one of the main signaling pathways mediating ERS response.

Similar studies in juvenile largemouth bass (*Micropterus salmoides*) and turbot (*Scophthalmus maximus*) demonstrated that high dietary carbohydrate levels induce the expression of *perk* and *grp78*, leading to ERS (Zhang et al. 2021; Zhao et al. 2021). Supplementation with taurine significantly reduced the increase in *perk* mRNA levels and GRP78 protein expression under high glucose conditions. This indicates that taurine may alleviate ERS induced by high glucose by suppressing the upregulation of ERS-related genes. This is consistent with the findings of our study, thus confirming the efficacy of taurine in alleviating ERS induced by high glucose.

### Effect of taurine on glycogen accumulation in GPM cells induced by high glucose

*Gysm* and *pygm* are reported as the two major enzymes affecting muscle glycogen metabolism (Furukawa et al. 2018). This study showed that high glucose treatment significantly increased the expression level of *gysm* in GPM cells and decreased the expression level of *pygm*. Additionally, high glucose treatment significantly increased the expression levels of GLUT4 and PGC-1α proteins, which play key roles in muscle glucose uptake (Richter and Hargreaves 2013). Compared to the high glucose group, taurine treatment restored the expression levels of *gysm* and *pygm* and significantly reduced the elevated expression levels of GLUT4 and PGC-1α proteins caused by high glucose. These results indicate that taurine supplementation improves the cell’s glucose uptake ability under high glucose conditions, regulating the balance of glycogen synthesis and breakdown, thereby effectively mitigating glycogen accumulation under high glucose conditions.

AMP-activated protein kinase (AMPK) is a cellular energy status sensor, with the ADP/ATP ratio positively correlating with AMPK activity. Studies have suggested that AMPK is activated under metabolic stress conditions, such as oxidative stress (Hardie et al. 2012). In studies on high glucose-induced Japanese flounder (*P. olivaceus*) muscle cells, the ADP/ATP ratio and ROS levels were significantly higher in the high glucose group, explaining the reason for the upregulated phosphorylation level of AMPK in this group (Liu et al. 2022a). In muscle cells of brown trout (*Salmo trutta*), activation of AMPK increased the mRNA levels of GLUT4, thereby enhancing glucose uptake (Díaz et al. 2007), suggesting that the activation of AMPK could be the primary reason for the enhanced glucose uptake in the high glucose group, mediated by a GLUT4-dependent mechanism. In this study, both the ADP/ATP ratio and ROS levels in the high glucose group were significantly higher compared to the control group, suggesting the activation of AMPK in the high glucose group. Although we did not directly measure AMPK phosphorylation, the elevated ADP/ATP ratio and ROS levels provide indirect evidence of AMPK activation, which has been well documented in other studies (Hardie et al. 2012). AMPK activation is known to influence several downstream pathways, including the PGC-1α pathway, which plays a critical role in regulating glycogen metabolism. Research has shown that PGC-1α is a key regulator of mitochondrial biogenesis and glucose metabolism (Liang et al. 2006). Upon AMPK activation, PGC-1α expression is enhanced, contributing to the regulation of muscle glycogen storage (Magnoni et al. 2012). In addition, PGC-1α has been shown to reduce the expression of *pygm*, further supporting its role in glycogen metabolism (Wenz et al. 2013). The interaction between AMPK and PGC-1α in the regulation of glucose uptake and glycogen storage under high glucose conditions suggests that taurine may alleviate glycogen accumulation by modulating the AMPK/PGC-1α pathway. Thus, taurine supplementation could help to restore the balance of glycogen synthesis and breakdown by activating AMPK and subsequently regulating PGC-1α, which enhances glucose uptake and improves muscle glycogen storage.

Moreover, our previous studies provide additional evidence on the beneficial role of taurine supplementation in *T. ovatus* subjected to high-carbohydrate, low-fishmeal diets. Specifically, Liu et al. (2022c) demonstrated that dietary taurine, at an optimal inclusion level of approximately 1.2% of the diet, significantly enhanced growth performance, improved antioxidant enzyme activities, and bolstered intestinal immune responses in *T. ovatus* fed high-carbohydrate diets. Furthermore, our work revealed that taurine supplementation modulated the endogenous taurine biosynthesis pathway, as indicated by the downregulation of key enzymes such as CDO and CSAD, suggesting a feedback mechanism that maintains taurine homeostasis under nutritional stress (Ma et al. 2021; Liang et al. 2024). Collectively, these findings not only corroborate the cellular-level protective effects observed in the present study but also underscore the potential application of taurine as an effective nutritional intervention to alleviate metabolic stress and improve overall fish health in aquaculture systems reliant on high-carbohydrate diets.

## Conclusion

**Fig. 7:**
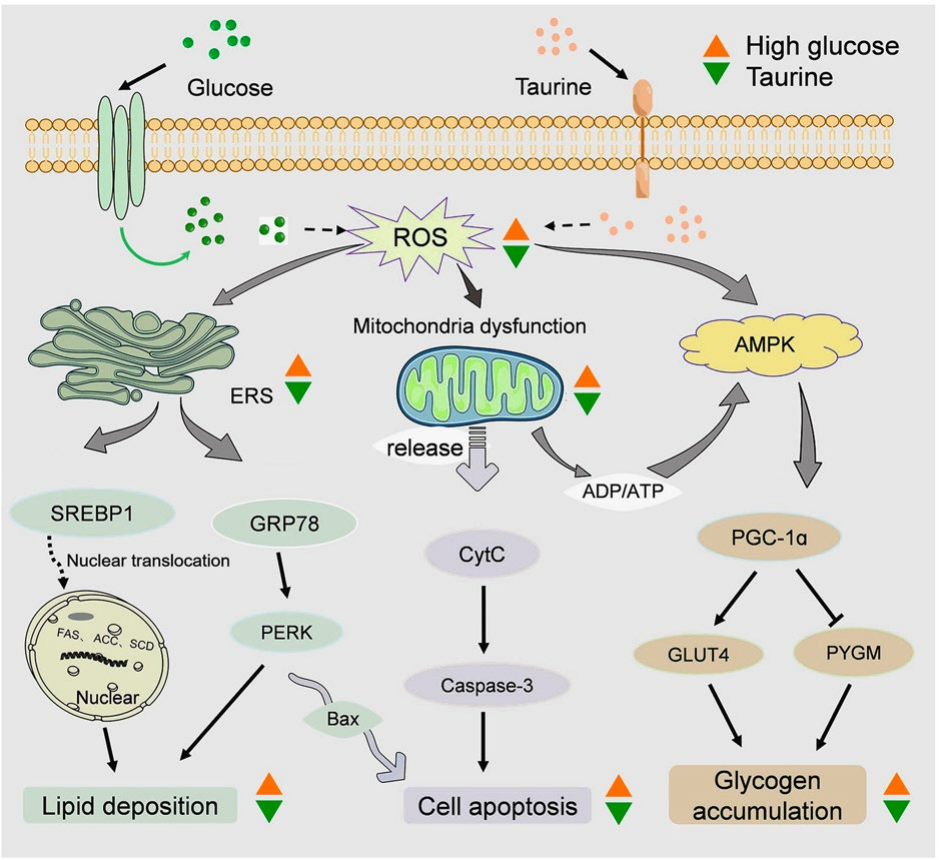
Regulatory mechanism pathways. The proposed mechanism of protective action of taurine against high glucose-induced apoptosis, lipid deposition, and glycogen accumulation in the muscle cells of golden pompano. Solid arrows represent stimulus-promoting effects, dotted lines represent possible target spot, and the “T” arrow represents inhibitory effect; the yellow triangle indicates increase and the green triangle indicates decrease

This study demonstrates that taurine mitigates the elevation of ROS and the activation of the AMPK/PGC-1α signaling pathway induced by high glucose. Additionally, taurine improves mitochondrial function under high glucose conditions and reduces the expression of genes related to glycolipid metabolism, apoptosis, and ERS. These findings indicate that taurine alleviates ERS, glycogen accumulation and mitochondrial oxidative stress caused by high glucose in the golden pompano muscle cells. Importantly, these results suggest that taurine supplementation could serve as an effective nutritional strategy in aquaculture practices, potentially enhancing fish health and muscle quality, reducing metabolic stress, and contributing to more sustainable and cost-effective production systems. The AMPK/PGC-1α signaling pathway, ERS response, and mitochondria-mediated apoptotic signaling pathways are involved in these biological effects (Fig. 7).

## Supplementary Information

Below is the link to the electronic supplementary material.

Supplementary file1 (DOCX 32 KB)

## Data Availability

The data presented in this study are being uploaded to the Genbank database.
