# Supplementary material for: Taurine alleviates endoplasmic reticulum stress, oxidative stress, apoptosis, and glycogen accumulation induced by high glucose in the muscle cells of golden pompano (Trachinotus ovatus)
Source: Mar Life Sci Technol. 2025 Oct 31;7(4):820–35. doi: 10.1007/s42995-025-00324-7 (PMC12662973; doi:10.1007/s42995-025-00324-7)
Supplement: Supplementary file 1 — Supplementary file1 (DOCX 32 KB) [file 42995_2025_324_MOESM1_ESM.docx]

**Taurine alleviates endoplasmic reticulum stress, oxidative stress, apoptosis and glycogen accumulation induced by high glucose in the muscle cells of** **golden pompano (*Trachinotus* *ovatus*)**

Ming-Jian Liu^1, 2 †^, Lu-Ke Zhang^1, 2 †^, Ke-Cheng Zhu^1, 2^, Hua-Yang Guo^1, 2^, Teng-Fei Zhu^1, 2^, Bao-Suo Liu^1, 2^, Nan Zhang^1, 2^, Dian-Chang Zhang^1, 2, 3 *^

^1^ Key Laboratory of South China Sea Fishery Resources Exploitation and Utilization, Ministry of Agriculture and Rural Affairs, South China Sea Fisheries Research Institute, Chinese Academy of Fishery Sciences, Guangzhou 510300, China

^2^ Sanya Tropical Fisheries Research Institute, Sanya 572019, China

^3^ Guangdong Provincial Engineer Technology Research Center of Marine Biological Seed Industry, Guangzhou 510300, China

† These authors contributed equally to this work.

* Correspondence:

Dian-Chang Zhang

Key Laboratory of South China Sea Fishery Resources Exploitation and Utilization, Ministry of Agriculture and Rural Affairs, South China Sea Fisheries Research Institute, Chinese Academy of Fishery Sciences, Guangzhou, 510300, Guangdong Province, China; Sanya Tropical Fisheries Research Institute, Sanya, Hainan Province, China

Tel: +862089108316; Fax: +862084451442

Email: zhangdch@scsfri.ac.cn

**The detailed experimental methods and operating steps are as follows.**

**1. Reactive oxygen species (ROS) detection and 4,6-diamino-2-phenyl indole (DAPI) staining (Detailed·description·of.2.3 .in the:main·text)**

This section involved the use of a ROS Assay Kit to determine the levels of reactive oxygen species within the cells. Cells in the logarithmic growth phase were evenly seeded at a density of 100,000 cells per well in a 6-well plate and incubated overnight at 28°C. The cells were then treated for 24 hours with Leibovitz's L-15 (L-15) complete medium containing 10% fetal bovine serum (FBS), L-15 medium with 60 mM glucose, and L-15 medium supplemented with 60 mM glucose and 15 mM taurine. These groups were named Control, HG (High Glucose), and HG + T (High Glucose + Taurine), respectively. After incubation, the ROS levels in each group were assessed.

(1) Following sufficient stimulation time, the cells were washed with phosphate-buffered saline (PBS).

(2) A mixture of DCFH-DA working solution and complete medium was added in a 1:1 ratio, totaling 2 ml. The cells were then incubated for 20 minutes.

(3) After the incubation period, cells were washed three times with L-15 medium to thoroughly remove any residual DCFH-DA probe.

(4) Cells were stained with DAPI nuclear staining solution, adding 1 mL of staining solution per well and incubating in the dark for 30 minutes, followed by three washes with 1 mL of PBS. An anti-fade agent was then added.

(5) The fluorescence of reactive oxygen species was observed under a fluorescence microscope using an excitation wavelength of 488 nm, and nuclear staining fluorescence was observed at an excitation wavelength of 364 nm.

**2. Mitochondrial membrane potential (MMP) and ADP/ATP ratio measurement (Detailed·description·of.2.4 .in the:main·text)**

Mitochondrial membrane potential changes in each group were detected using a Mitochondrial Membrane Potential Assay Kit. Cells were treated for 24 h under the same conditions as described above. Following incubation, the culture medium was discarded, and 500 µL of JC-1 working solution was added, followed by a 30-minute incubation at 28°C. Cells were then extracted, washed twice with staining buffer, and replenished with complete medium for fluorescence intensity observation under a fluorescence microscope.

The ADP/ATP ratio in each group was measured using an ADP/ATP Ratio Assay Kit (bioluminescence method). After 24 hours of incubation under the specified conditions, cells were processed according to the kit's instructions. The ATP reaction mixture was added and incubated for 2 minutes, followed by ATP analysis using a luminometer. Subsequently, the prepared ADP reaction mixture was added to the same wells and incubated for another 2 minutes, after which ADP was analyzed using the luminometer. The ADP/ATP ratio was then calculated.

**3. Cell apoptosis detection (Detailed·description·of.2.5 .in the:main·text)**

Cell apoptosis in each group was detected using an AnnexinV-FITC/PI Double Staining Cell Apoptosis Detection Kit. The cell treatment method was consistent with the previous steps, followed by cell collection 24 hours later and three washes with PBS.

(1) After terminating digestion, the cells were uniformly resuspended in the culture medium and centrifuged at 600 g for 3 minutes. The supernatant was discarded, and the cells were collected, washed again with PBS, and centrifuged, discarding the supernatant and retaining the cell pellet.

(2) An appropriate amount of binding buffer was added, and cells were gently resuspended using a pipette to achieve a concentration of 1×10^6 cells/mL.

(3) 100 µL of the cell suspension was transferred to another centrifuge tube, and 10 µL of pre-mixed AnnexinV-FITC and PI staining solution was added. The mixture was incubated for 15 minutes at room temperature in the dark.

(4) 400 µL of 1× Binding Buffer was added to each tube, and flow cytometric analysis was performed, selecting appropriate channels for detection (FITC fluorescence was detected in the FL1 channel, and PI fluorescence was detected in the FL3 or FL2 channel).

**4. Caspase-3 activity measurement (Detailed·description·of.2.6 .in the:main·text)**

Caspase-3 activity was determined using a Caspase-3 Activity Assay Kit. The cell treatment method was consistent with previous descriptions, with a 24-hour treatment according to the conditions of each group. The procedure was as follows:

(1) Discard the culture medium and wash the cell surface with phosphate-buffered saline (PBS) to remove residual substances. Terminate digestion and centrifuge the cells, then discard the supernatant.

(2) Add lysis buffer to the centrifuge tube containing the cell pellet and perform low-temperature lysis on ice.

(3) Centrifuge at 12,000 rpm for 10-15 minutes at 4°C and collect the supernatant.

(4) Determine protein concentration using the Bradford Protein Assay Kit.

(5) Take 50 µL of the sample, add Ac-DEVD-pNA, mix well, and incubate at 28°C for 4 hours.

(6) Observe periodically during incubation; once a significant color change occurs, measure the absorbance at 405 nm using a spectrophotometer to calculate the activation level of caspase-3.

**5. Glycogen content analysis and Periodic Acid-Schiff (PAS) staining (Detailed·description·of.2.7 .in the:main·text)**

This section involved using a Glycogen Content Assay Kit and Glycogen PAS Staining Kit. The cell treatment method was the same as above, with a 24-hour treatment according to each group's conditions. The glycogen content and PAS staining results of each group were measured following the manufacturer's instructions.

(1) Glycogen content measurement

After digestion with trypsin and low-speed centrifugation for 3 minutes, cell pellets were collected and lysed with 0.75 mL of extraction solution using ultrasonication. The lysates were then boiled in a water bath for 20 minutes, shaking intermittently for uniform mixing. Next, the volume was made up to 5 mL with distilled water, mixed well, and centrifuged at high speed for 10 minutes. The supernatant was used for measurement. 200 µL of the supernatant was added to a 96-well plate, and the absorbance at 620 nm was measured using a spectrophotometer to determine the glycogen content of each group.

(2) Glycogen staining

a) Discard the supernatant and wash the cells twice with PBS, then fix with PAS fixing solution for 10 minutes.

b) Discard the fixing solution, rinse with water, and air-dry.

c) Add glycogen oxidant and react at room temperature for 15-20 minutes. Rinse twice with tap water and twice with distilled water.

d) Immerse in Schiff's reagent in the dark for 10-20 minutes.

e) Rinse with running water for 5 minutes.

f) Add Mayer's hematoxylin for counterstaining for 1-2 minutes. Discard the stain and rinse 2-5 times with water.

g) Air-dry and photograph.

**6. Nile Red and DAPI staining (Detailed·description·of.2.8 .in the:main·text)**

Nile Red staining was used to detect intracellular lipid content using a Nile Red Staining Kit. The cell treatment method was consistent with the previous steps, with a 24-hour treatment according to the conditions of each group. After incubation, cells were gently washed twice with phosphate-buffered saline (PBS) and fixed with 500 µL of 4% paraformaldehyde for 10 minutes. The fixative was then removed, and cells were washed twice with PBS. Next, each well received 500 µL of Nile Red staining solution at a concentration of 1 mmol/L, with a staining duration of 10 minutes. After discarding the Nile Red staining solution, 500 µL of DAPI staining solution at a concentration of 1 µg/mL was added to each well for 10 minutes. The DAPI staining solution was then discarded, and 500 µL of PBS was added to each well. Observations and photographs were taken under an inverted fluorescence microscope.

**7. Triglyceride content measurement (Detailed·description·of.2.9 .in the:main·text)**

Triglyceride (TG) content was measured using a Triglyceride Assay Kit. The cell treatment method was the same as above, with a 24-hour treatment according to each group's conditions. After incubation, the culture medium was discarded, and 30 µL of 1% to 2% lysis buffer was added to each well. Cells were carefully scraped from the plate and transferred to a 1.5 mL Eppendorf tube for lysis on ice for 20-30 minutes. After centrifugation, 2 µL of the supernatant was taken to measure protein concentration using a Total Protein (TP) Assay Kit. The remaining supernatant was placed in a 70°C oven for 10 minutes, followed by centrifugation at 600 g for 5 minutes. The supernatant was then used for measuring intracellular TG content. Samples were prepared according to the kit instructions, mixed well, and 200 µL was added to a 96-well plate. The absorbance at 550 nm was measured using a spectrophotometer to determine the TG content of each group.

**8. Observation of ER and mitochondrial ultrastructure in GPM cells (Detailed·description·of.2.10 .in the:main·text)**

Cells were plated at a density of 50,000 cells per well and treated for 24 hours according to the conditions of each group. After incubation, cells were digested with trypsin, centrifuged to discard the supernatant, and the cell pellet was processed for transmission electron microscopy to observe the ultrastructure of golden pompano muscle cells. The procedure was as follows: cell pellets from each group were fixed overnight with 2.5% glutaraldehyde, washed three times with PBS for 10 minutes each, and then fixed with 2% osmium tetroxide for 2.5 hours. Graded ethanol dehydration was performed, followed by three 30-minute acetone replacements. Cells were then infiltrated with embedding medium for 4 hours and placed in pure embedding medium in embedding molds, polymerized at 40°C and 60°C for 48 hours each. Ultrathin sections were prepared, stained with uranyl acetate and lead citrate for 5-15 minutes, washed, and then observed under a transmission electron microscope for the ultrastructure of the ER and mitochondria.

**9. Enzyme activity measurement of glycolipid metabolism (Detailed·description·of.2.11 .in the:main·text)**

Cells, adjusted to a density of 50,000 cells per well, were plated and treated for 24 hours according to the respective group conditions. After incubation, cells from each group were digested and counted under a microscope. An appropriate amount of extraction liquid, as per the instruction manual, was added to collect the cells into Eppendorf tubes for ultrasonic disruption. The samples were then centrifuged at high speed for 10 minutes (12,000 × g, 4°C), and the supernatant was collected and placed on ice for analysis.

To investigate the impact of high glucose on the sugar and lipid metabolism capabilities of golden pompano (*Trachinotus ovatus*) muscle cells, this study measured the enzyme activities of acetyl-CoA carboxylase (ACC), fatty acid synthase (FAS), hormone-sensitive lipase (HSL), adipose triglyceride lipase (ATGL), glucose-6-phosphate dehydrogenase (G6PD), malic enzyme (ME), glycogen synthase (GYSM), and glycogen phosphorylase (PYGM) in these cells. Biochemical assay kits for these enzymes were provided by Jiangsu Enzyme Immune Industrial Co., Ltd.

According to the kit instructions, the initial enzyme activity results were expressed in U/L. In our experiment, each well contained approximately 5×10^^4^ cells, and 1 mL of cell lysate was used for the assay. To normalize the data to cell number, we multiplied the measured U/L by the assay volume (L) to obtain the total enzyme activity (U), then divided by the total cell count for each sample and multiplied by 10^^6^, ultimately expressing the results as U/10^^6^ cells.

**10. Real-Time quantitative PCR for mRNA expression levels (Detailed·description·of.2.12 .in the:main·text)**

Cells were plated at a density of 50,000 cells per well and treated according to the conditions for each group for 24 hours. After incubation, cells from each well were digested with trypsin, digestion was terminated with complete medium, and cells were centrifuged to discard the supernatant.

Total RNA from GPM cells was extracted using the HiPure Universal RNA Kit following the manufacturer's instructions, and RNA concentration along with the OD260/OD280 ratio was assessed using NanoDrop2000 and 1% agarose gel electrophoresis. cDNA synthesis was performed using the Evo M-MLV RT Mix Kit with gDNA Clean for qPCR, and gene expression was quantified on the Roche LightCycler® 480 II system using the SYBR Green Premix Pro Taq HS qPCR Kit. Primers for GRP78, PERK, SREBP1, SCD, FAS, ACC, HSL, LPL, OPA1, MFN2, GYSM, and PYGM were designed using Primer Premier 6.0, with EF-1α serving as the internal reference gene. Each sample was run in triplicate, and the amplification and melting curves were confirmed after RT-qPCR to ensure specificity. Gene expression was analyzed using the EF-1α as a reference gene and the 2^-ΔΔCT^ method.

(1) Total RNA extraction

a) Disperse cell clumps with 1 mL of lysis buffer, thoroughly mix for complete lysis, and incubate at room temperature for 5-10 minutes.

b) After incubation, add 200 μL chloroform, vigorously shake for 15 seconds, incubate at room temperature for 3 minutes, and then centrifuge at 12,000 × g for 15 minutes at 4°C for phase separation.

c) Transfer 400 μL of the supernatant to a new 1.5 mL centrifuge tube and mix with 1.5 times the volume of anhydrous ethanol.

d) Transfer the mixture to a 2 mL collection tube containing a column and centrifuge at 12,000 g for 30-60 seconds until all the liquid passes through the column.

e) Discard the flow-through, add 600 μL Buffer RW2 to the column, and centrifuge at 12,000 g/min for 1 minute at 4°C; this step can be repeated twice.

f) Discard the flow-through and centrifuge the empty column. Transfer the column to a new centrifuge tube, add 70 μL RNase-Free Water to the center of the column membrane, let stand for 2 minutes, and then centrifuge. A second elution can be chosen.

g) Store the RNA at -80°C in the centrifuge tube.

(2) cDNA synthesis

1) Remove genomic DNA

| Reagent name | Dosage |
| --- | --- |
| 5×gDNA Clean Reaction Mix | 2 μL |
| Total RNA | 1 μg |
| RNase free water | up to 10 μL |

Reaction conditions：

42℃ 2 min

4℃ ∞

2) Reverse transcription reaction

| Reagent name | Dosage |
| --- | --- |
| Step 1) Reaction solution | 10 μL |
| 5×*Evo* *M*-*MLV* RT Reaction Mix | 4 μL |
| RNase free water | 6 μL |
| Total | 20 μL |

Reaction conditions：

37℃ 15 min

85℃ 5 s

4℃ ∞

After the reaction, the cDNA was stored at -20°C until use.

(3) RT-qPCR reaction

| Reagent name | Dosage |
| --- | --- |
| 2 × SYBR Green Pro Taq HS Premix | 6.25 µL |
| ddH_2_O | 4.25 µL |
| forward primer | 0.5 μL |
| reverse primer | 0.5 μL |
| cDNA template | 1 μL |

Reaction conditions:

95℃ 30 s

95℃ 10 s

60℃ 30 s

72℃ 30 s

40个cycle

After the reaction, the 2^-ΔΔCT^ method was used for data analysis.

(4) Primer sequences used in the experiment

| Primer name | Forward primer sequence (5′-3′) | Reverse primer sequence (5′-3′) |
| --- | --- | --- |
| *GRP78* | CCCCTGACTCTTGGTATTG | CTTGTTCTTGTTGCCTGTG |
| *PERK* | AGTCCTTGCTGGAGTCGT | TTCGGTTCCTCTGTGATG |
| *FAS* | GATGGATACAAAGAGCAAGG | GTGGAGCCGATAAGAAGA |
| *SCD* | GGCTACCGCAACAAGTC | GTGTGAAGTAATCCACCAAAT |
| *ACC* | GTTGTCAATCCCAGCCGATC | ATCCACAATGTAGGCCCCAA |
| *SREBP1* | GAGCCAAGACAGAGGAGTGT | GTCCTCTTGTCTCCCAGCTT |
| *HSL* | TCATACCTCCACACCAACCC | GTCTCGCAGTTTCTTGGCAA |
| *LPL* | TTTGTCCTTCCTCGTCACCA | AAGACAGCATCCTCTCCACC |
| *OPA1* | AGCAGAATCCAGCAAATA | GCTCAACAGACTCCCTAAC |
| *MFN2* | CTGGTCAACCGCTTCCT | GCTGGCGTAGTCCACAAA |
| *GYSM* | CTTACTATGAGCCTTGGGG | GCCGAGGTATCTCCAGTC |
| *PYGM* | CAACCGCATCAAGAAGG | TCAGTGCCTGCTGTGGA |
| *EF*-*1α* | AAGCCAGGTATGGTTGTCAACTTT | CGTGGTGCATCTCCACAGACT |

**11. Western blot for protein expression analysis (Detailed·description·of.2.13 .in the:main·text)**

(1) Extraction and preservation of total cellular proteins

a) Cell Preparation: Logarithmically growing cells were evenly plated at a density of 100,000 cells per well in a 6-well plate and incubated overnight at 28°C. Cells were then treated according to the respective group conditions for 24 hours.

b) Protein Extraction: Cell culture medium was aspirated, and 80 µL of lysis buffer was added to each well. The plates were placed at 4°C for 10 minutes to allow complete lysis. The lysate was then transferred to centrifuge tubes and centrifuged at 12,000 × g for 20 minutes at 4°C. The supernatant was collected for total protein extraction and immediately quantified.

c) Protein Quantification: Quantification was carried out using the BCA Protein Assay Kit as follows:

i. 30 mg of standard protein was dissolved in 1.2 mL of dilution buffer to prepare a 25 mg/mL standard solution. This solution was further diluted to 0.5 mg/mL with PBS.

ii. Reagent A and Reagent B were mixed in a 550:11 volume ratio to prepare the BCA working solution.

iii. The standard solution was added to a 96-well plate in different dilutions alongside the protein samples in triplicates.

iv. 200 µL of BCA working solution was added to each well, and the plate was incubated at 37°C for 30 minutes.

v. Absorbance at 562 nm was measured, and a standard curve was plotted to calculate the regression equation.

d) Preservation of Total Protein: The extracted proteins were mixed with 5× SDS loading buffer (1:4 ratio), boiled at 100°C for 10 minutes, and stored at -20°C for future use.

Antibody Information Used in Western Blot Analysis

| Reagent name | Manufacturers | Catalog |
| --- | --- | --- |
| Bax | HUABIO | ET1603-34 |
| Bcl-2 | HUABIO | ET1705-45 |
| CytC | HUABIO | ET1610-60 |
| GRP78 | HUABIO | ET1602-39 |
| PGC-1α | HUABIO | ER40402 |
| GLUT4 | HUABIO | R1402-3 |
| EF | HUABIO | ER65602 |

(2) Western blot analysis

a) SDS-PAGE Preparation: Two glass plates were placed into a glass plate holder, aligned, and clamped. The holder was fixed vertically on a stand. Separating gel was poured between the glass plates, followed by overlaying with DEPC water. Once the gel had solidified, the water was discarded and the stacking gel was poured in. A comb was inserted to create wells and removed after the gel solidified. The gel was then placed in an electrophoresis tank and filled with running buffer.

b) Electrophoresis: 30 µg of protein samples from each group were loaded into the wells. Initial electrophoresis was performed on the stacking gel at 80 V for 20 minutes, followed by the separating gel at 120 V for 60 minutes. Electrophoresis was stopped once the marker reached the appropriate position, and the gel was prepared for transfer.

c) Transfer: The PVDF membrane was activated in methanol for 5 minutes and cut to the size of the gel. The gel, along with filter paper and sponge, was soaked in transfer buffer. The assembled sandwich was placed in the transfer apparatus and transferred at 200 mA for 90 minutes. The membrane was then blocked in blocking solution at 37°C for 1 hour, followed by overnight incubation at 4°C with primary antibodies. The membrane was washed four times with TBST for 5 minutes each, then incubated with secondary antibodies at 37°C for 1 hour, followed by another five 5-minute washes in TBST.

d) Detection: The ECL detection method was used as per the manufacturer's instructions. ECL solution was applied to the membrane for 1-2 minutes, then imaged after discarding the solution, adjusting exposure time as necessary to detect bands.

**12. List of Reagents and Corresponding Details Used in the Experiment (Detailed·description·of.2.14 .in the:main·text)**

1. **Leibovitz's L-15 (Leibovitz's L-15 complete medium, Gibco, Cat. No. 11415064, Carlsbad, CA, USA)**
2. **Fetal Bovine Serum (FBS, Gibco, Cat. No. 10099-141, Carlsbad, CA, USA)**
3. **Phosphate-Buffered Saline (PBS, Goodbio, Cat. No. G4202, Shanghai, China)**
4. Glucose (D-Glucose, Sigma-Aldrich, Cat. No. G8769, ≥99% purity)
5. **Trypsin (Trypsin, Gibco, Cat. No. 25200056, Carlsbad, CA, USA)**
6. **D-Glucose (D-Glucose, Sigma-Aldrich, Cat. No. G8769, ≥99% purity, St. Louis, MO, USA)**
7. **Taurine (Taurine, Sigma-Aldrich, Cat. No. T0625, ≥98% purity, St. Louis, MO, USA)**
8. **Cell Counting Kit-8 (CCK-8, Shanghai Yuanye Bio-technology Co. Ltd, Shanghai, China)**
9. **Reactive Oxygen Species Detection Kit (ROS Assay Kit, Gibco, Cat. No. C10444, Carlsbad, CA, USA)**
10. **2’-7’ Dichlorofluorescin Diacetate (DCFH-DA, Sigma-Aldrich, Cat. No. D6883, St. Louis, MO, USA)**
11. **DAPI Staining Solution (DAPI staining solution, Gibco, Cat. No. D1306, Thermo Fisher Scientific, Carlsbad, CA, USA)**
12. **MitoProbe™ JC-1 Assay Kit (MitoProbe™ JC-1 Assay Kit, Invitrogen™, Cat. No. M34152, Carlsbad, CA, USA)**
13. **ADP/ATP Ratio Assay Kit (ADP/ATP Ratio Assay Kit, Sigma-Aldrich, Cat. No. MAK135-1KT, St. Louis, MO, USA)**
14. **Annexin V-FITC Apoptosis Detection Kit (Annexin V-FITC Apoptosis Detection Kit, Beyotime, Cat. No. C1063, Shanghai, China)**
15. **Caspase-3 Activity Assay Kit (EnzChek™ Caspase-3 Activity Assay Kit, Invitrogen™, Cat. No. E13184, Thermo Fisher Scientific, Carlsbad, CA, USA)**
16. **Bradford Protein Assay Kit (Bradford Protein Assay Kit, Beyotime, Cat. No. P0006, Shanghai, China)**
17. **Glycogen Content Assay Kit (Glycogen Content Assay Kit, Abcam, Cat. No. ab65620, Cambridge, MA, USA)**
18. **PAS Staining Kit (PAS Staining Kit, Abcam, Cat. No. ab150680, Cambridge, MA, USA)**
19. **Nile Red Staining Kit (Nile Red Staining Kit, Sigma-Aldrich, Cat. No. N3013, St. Louis, MO, USA)**
20. **Triglyceride Assay Kit (Triglyceride Assay Kit, Sigma-Aldrich, Cat. No. MAK266, St. Louis, MO, USA)**
21. **Transmission Electron Microscopy (TEM) related reagents (Custom experimental methods, no specific brand or catalog number provided)**
22. **HiPure RNA Extraction Kit (HiPure Kit, Magen, Cat. No. R4121-02B, China)**
23. **Evo M-MLV RT Mix Kit (Evo M-MLV RT Mix Kit, Accurate Biotechnology, Cat. No. AG11728, China)**
24. **gDNA Clean for qPCR (gDNA Clean for qPCR, Accurate Biotechnology, Cat. No. AG11701, China)**
25. Thermo Scientific Mem-PER Plus Membrane Protein Extraction Kit (Thermo Scientific, Cat. No. 89842)
26. **SDS-PAGE Electrophoresis Reagents (General laboratory supplies, no specific brand or catalog number provided)**
27. **Pierce BCA Protein Assay Kit (Pierce BCA Protein Assay Kit, Thermo Scientific, Cat. No. 23227, Carlsbad, CA, USA)**
28. **Western Blot ECL Detection Reagents (Custom experimental methods, no specific brand or catalog number provided)**
29. **Flow Cytometry reagents (Antibodies and reagents used for flow cytometry, general suppliers based on laboratory preferences, no specific brand provided)**
